# Measuring evolutionary rates of proteins in a structural context

**DOI:** 10.12688/f1000research.12874.2

**Published:** 2018-02-09

**Authors:** Dariya K. Sydykova, Benjamin R. Jack, Stephanie J. Spielman, Claus O. Wilke

**Affiliations:** 1 Department of Integrative Biology, The University of Texas at Austin, Austin, TX, 78712, USA; 2 Institute for Genomics and Evolutionary Medicine, Temple University, Philadelphia, PA, 19122, USA

**Keywords:** Protein evolution, protein structure, evolutionary rate, relative solvent accessibility, weighted contact number, multiple sequence alignment

## Abstract

We describe how to measure site-specific rates of evolution in protein-coding genes and how to correlate these rates with structural features of the expressed protein, such as relative solvent accessibility, secondary structure, or weighted contact number. We present two alternative approaches to rate calculations: One based on relative amino-acid rates, and the other based on site-specific codon rates measured as *dN*/ *dS*. We additionally provide a code repository containing scripts to facilitate the specific analysis protocols we recommend.

## Introduction

Different sites within a protein-coding gene evolve at different rates ^1, 2^. This evolutionary rate heterogeneity across protein sites results from a complex interplay of both functional and structural constraints ^3^. For example, residues that are critical to a given protein’s function, such as residues involved in enzymatic activity, protein–protein interactions, or protein–ligand interactions, tend to evolve more slowly than do other residues in the protein ^4– 10^. In addition, a given protein’s structure plays a major role in shaping its evolutionary rates, due to the overarching evolutionary constraint exerted by the imperative for a protein to stably fold. Structurally-important protein residues (namely residues in the protein core) tend to be highly conserved and evolve very slowly, whereas residues with a relatively minor influence on structure (namely surface residues) tend to evolve more rapidly ^4, 9, 11– 19^.

To study evolutionary conservation in a structural context, we require methods to (i) measure evolutionary rates at individual sites in a protein alignment, (ii) map those rates onto the protein structure, and (iii) quantify site-level structural properties. Here, we describe in detail how to perform these three steps, considering a few commonly used alternatives at both steps (i) and (iii). In addition, we provide extensive notes highlighting specific technical issues and/or describing alternative analysis approaches.

At step (i), we demonstrate how evolutionary rates can be measured using either amino-acid or codon data. For amino-acid data, we consider relative amino-acid rates, i.e., rates of evolutionary variation normalized by the mean of the rate in the entire protein ^10, 20^. For codon data, we consider site-specific *dN/dS *values. These are site-specific rates of nonsynonymous variation normalized by (in this case, the whole-gene) rates of synonymous variation ^21, 22^.

At step (iii), we discuss two related but somewhat distinct structural measures. First, we consider the solvent accessibility, which measures the extend to which a site is exposed to the solvent environment. Specifically, we consider the relative solvent accessibility (RSA) ^23^, which is the solvent accessibility of a residue in a structure normalized by the maximum possible solvent accessibility of that residue in a Gly-X-Gly tripeptide. Second, we consider the packing density, which measures the proximity to and number of neighboring residues. Specifically, we consider the side-chain weighted contact number (WCN) ^19^, which is calculated relative to the geometric center of the residue side-chain atoms and employs an inverse-square weighting term.

## Materials

Below we list the software packages needed to perform the analysis. Please download the most recent version of each software, unless a specific version is specified in the text. The links provided contain instructions for installing and testing the software. All analyses we present assume that these software packages have been installed and are available in your path.

1. HyPhy ( *see* **Note 1**)
  HyPhy is a general-purpose software platform for inference in a phylogenetic framework ^24^. To install, either clone the HyPhy git repository to your desired directory, or download the latest release. The HyPhy repository can be found at https://github.com/veg/hyphy.git. Follow the instructions available from http://hyphy.org/installation to install HyPhy. Importantly, ensure that you are installing version 2.3.8 or above.
2. MAFFT
  MAFFT is a program for generating multiple sequence alignments ^25^. Download MAFFT from http://mafft.cbrc.jp/alignment/software/.
3. RAxML
  RAxML is a tool for phylogenetic inference using maximum likelihood ^26^. Clone the RAxML repository to a local directory. The RAxML git repository can be found at https://github.com/stamatak/standard-RAxML. Analyses presented here utilize the raxmlHPC-SSE3 executable, which can be compiled with Makefile.SSE3.gcc or Makefile.SSE3.mac. Note that this executable does not allow threading. ( *See* **Note 2** for information on how to enable threading.)
4. mkDSSP
  mkDSSP is a tool that calculates solvent accessibilities and parses secondary structure assignments from a PDB input file into a standardized format ^27^. This format follows that of the entries in the DSSP database ^28^. Download the mkDSSP software from https://slackbuilds.org/repository/14.2/academic/mkDSSP/.
5. Python
  Download python from https://www.python.org/downloads/.
6. Biopython
  Biopython is a python library for computational molecular biology ^29^. Download Biopython from http://biopython.org/wiki/Download.
  Biopython has several dependencies that also need to be installed. You can find the information about installing the dependencies in the link provided.
7. argparse
  argparse is a python module providing user-friendly command-line interfaces. We use argparse in most of our custom python scripts. Install argparse using the link https://pypi.python.org/pypi/argparse.
8. pandas
  pandas is a python module for data manipulation and analysis. You can download pandas from https://pandas.pydata.org/getpandas.html.
9. R
  Download R from https://www.r-project.org/. We recommend to use RStudio to execute and edit R scripts. RStudio can be installed from https://www.rstudio.com/. We will use R for data visualization. Our scripts require the packages dplyr, readr, cowplot, and their dependencies. You can install an R package by typing the command install.packages("dplyr") (for installing dplyr) in the R shell. By default, this command will also install any dependencies needed for the package to work.
10. Custom scripts ( *see* **Note 3**)
  All our custom python, R, and HyPhy scripts can be found at:
https://github.com/clauswilke/proteinER/tree/master/src.

## Protocols

In this section, we provide four separate protocols to (i) measure relative amino acid rates, (ii) measure site-specific codon evolutionary rates (expressed via the metric *dN/dS*), (iii) measure structural quantities such as RSA and WCN, and (iv) combine the measured quantities into a combined analysis. To provide an example, we demonstrate all four protocols on an empirical dataset consisting of mammalian orthologs of histamine receptor 1 (HRH1; ENST00000438284) and an accompanying HRH1 PDB structure, 3rze ^30^. This dataset was originally analyzed by Spielman and Wilke ^31^.

Throughout, we assume that we are working on UNIX-like command line interface. We recommend that a user is comfortable with command execution and syntax, which includes flags, arguments, and directories. No prior knowledge of any of the listed software is essential. For python and R scripts, we provide detailed description for each script’s function. As such, it is not strictly necessary that a user knows python or R to execute our pipeline. However, if more detailed understanding of the custom scripts is desired, a user should be familiar with python and R. For your convenience, we have provided a git repository at https://github.com/clauswilke/proteinER/ that contains the input and output files used in each step.

Our overarching strategy throughout this work is to first infer a given measurement (e.g., *dN/dS* or RSA) for each site in the multiple sequence alignment or protein structure. To compare the different measurements, we then map them all to columns in the multiple sequence alignment.

### Protocol 1: Measuring relative amino-acid rates

The input and output files used in this section can be found at: https://github.com/clauswilke/proteinER/tree/master/measuring_aa_rates.

1. Collect amino acid sequences
  One of the most popular methods to collect sequences is BLAST ^32, 33^. To search for orthologous sequences in the NCBI database, determine a query sequence, on which BLAST will base its homology search. The BLAST output will specify the number and percent of sites with matches, near matches, and no matches. BLAST refers to these as identities, positives, and gaps, respectfully. We recommend the specific algorithm PSI-BLAST ^33^ if one is interested in collected amino-acid sequences (as opposed to nucleotide). For either data type, we recommend that users specify that BLAST query NCBI “RefSeq” (reference sequence) ^34^, which has been heavily curated to contain only nonredundant and reliably annotated sequences. Aside from BLAST, many other approaches, including collecting orthologs from databases such as ENSEMBL ^35^ or UniProt ^36^, are also suitable for this step. Regardless of the approach taken, we recommend that a final dataset contain at least 20 sequences, with more being preferable, to achieve reliable evolutionary rate estimates.
2. Align sequences with MAFFT ( *see* **Note 4**)
  Store all of the sequences you wish to align into one FASTA-formatted file. The FASTA format contains two pieces of information for each sequence: the sequence ID preceded by a ">" sign and followed by a new line, and then the sequence itself. We will use the FASTA file HRH1_unaligned.fasta that contains homologous sequences that are not aligned. We align them with the command:

mafft --auto --inputorder \
       
                                HRH1_unaligned.fasta > \
       
                                HRH1_aligned.fasta
  Arguments above correspond to the following:
  • --auto, Select the optimal alignment algorithm for the given data.
  • --inputorder, Output sequences in the same order in which they were provided. Without this option, the order of the sequences in the alignment is arbitrary.
  The output file HRH1_aligned.fasta will contain the aligned sequences.
3. Infer tree with RAxML ( *see* **Notes 2, 5**)
  Using the file with the alignment HRH1_aligned.fasta, run RAxML with the following command:

raxmlHPC-SSE3 -s HRH1_aligned.fasta \
                
                                -n HRH1_tree \
                
                                -m PROTCATLG \
                
                                -p 12345
  Arguments above correspond to the following:
  • -s, The multiple sequence alignment file.
  • -n, The extension for the outputted tree files. Here, the outputted files will contain HRH1_tree in their names.
  • -m, The model of sequence evolution, in this case the LG amino-acid model ^37^ with RAxML’s “CAT" model ^38^ of sequence heterogeneity.
  • -p, The random number seed initializing this phylogenetic inference. To reproduce the exact phylogeny we have, specify this random seed.
  The desired tree file is RAxML_bestTree.HRH1_tree.
4. Infer site-wise rates with HyPhy ( *see* **Note 6**)
  We calculate rates with the LEISR method in HyPhy ^39^. To run this method, the file runLEISR.bf must be edited to specify the directories and file names that will be used in the analysis. Edit these two lines of runLEISR.bf

"0": "/path/to/HRH1_aligned.fasta",

                                "1": "/path/to/RAxML_bestTree.HRH1_tree",

  Here, "0" should specify the full path to the alignment file HRH1_aligned.fasta, and "1" should specify the full path to the tree file RAxML_bestTree.HRH1_tree.
  Run HyPhy with the command
HYPHYMP runLEISR.bf
  An output file HRH1_aligned.fasta.LEISR.json is written to the folder that contains the alignment.
5. Parse HyPhy output ( *see* **Note 3**)
  For further downstream processing, the HyPhy output file in JSON format needs to be converted to CSV format. The custom python script parse_LEISR.py will extract each site’s position, rate, and other site-specific inference information from the JSON output file. Parse the JSON file with the command

python parse_LEISR.py \

                                -j HRH1_aligned.fasta.LEISR.json \

                                -r extracted_HRH1_rates.csv
  Arguments above correspond to the following:
  • -j, JSON file outputted by HyPhy.
  • -r, The output CSV file. If not specified, the output file is site_rates.csv.
6. Calculate relative site-wise rates ( *see* **Note 7**)
  As discussed by Jack *et al*. ^10^, we recommend calculating relative evolutionary rates by normalizing inferred site-specific rates by their average. In other words, to compute the relative amino-acid rates, calculate the mean rate of the entire sequence and divide each site’s rate by this mean rate. Once normalized, a rate below 1 will indicate a site that evolves more slowly than average. For example, a rate of 0.5 implies that the corresponding site evolves half as quickly as does the average. Similarly, a rate above 1 will indicate a site that evolve more quickly than average. For example, a rate of 2 implies that the corresponding site evolves twice as quickly as does the average.

### Protocol 2: Measuring site-specific *dN/dS*

The input and output files used in this section can be found at: https://github.com/clauswilke/proteinER/tree/master/measuring_dNdS.

1. Collect nucleotide sequences
Collect nucleotide sequence using step 1 in Protocol 1, using a nucleotide sequence as the query. Alternatively, the Ensembl database’s Biomart tool ^35^ may represent a more reliable approach for collecting strictly protein-coding sequences. By contrast, the UniProt database ^36^ should not be used, as it contains only protein sequences and lacks clear cross-references to the corresponding nucleotide sequences.
2. Translate codon sequences ( *see* **Note 3**)
  In this section, both codon and amino-acid sequences are required to perform site-wise rate calculations. Store all of the desired nucleotide sequences into one FASTA file. Use our custom script to convert a codon FASTA file to an amino acid FASTA files. We use the FASTA file HRH1_unaligned_codon.fasta that contains homologous nucleotide sequences we wish to translate. Translate with the command:

python translate_aln_codon_to_aa.py \
        
                                -n HRH1_unaligned_codon.fasta \
        
                                -o HRH1_unaligned_aa.fasta

  Arguments above correspond to the following:
  • -n, The input file with codon sequences. Both aligned and unaligned sequences are accepted.
  • -o, The output file with amino acid sequences. If not specified, the script outputs aa_aln.fasta. If the input file contains aligned sequences, the output file will also contain aligned sequences.
3. Align amino acid sequences with MAFFT
Align amino acid sequences using step 2 in Protocol 1.
4. Back-translate the amino acid alignment into a codon alignment ( *see* **Note 3**)
This step requires the original codon sequences and the amino acid alignment. Note that the amino acid alignment is retained, and the script simply inserts corresponding codons in place of amino acids at each column of the alignment. Use this command to back-translate the sequences:

python translate_aln_aa_to_codon.py \
         
                                -a HRH1_aligned_aa.fasta \
         
                                -n HRH1_unaligned_codon.fasta \
         
                                -o HRH1_aligned_codon.fasta
  Arguments above correspond to the following:
  • -a, The inputted amino-acid alignment.
  • -n, The file of codon sequences. The script accepts either aligned or unaligned sequences.
  • -o, The output file to contain the codon alignment. This argument is optional, and, if it is missing, the script outputs a file codon_aln.fasta.
5. Infer tree with RAxML
  The following step is the same as step 3 in Protocol 1. Use the amino-acid alignment file HRH1_aligned_aa.fasta to infer the tree.
6. Infer site-wise rates with HyPhy ( *see* **Note 8**)
  To calculate site-wise *dN/dS*, we use the Fixed Effects Likelihood (FEL) method in HyPhy ^21^. To run FEL in HyPhy, the file runFEL.bf must be edited to specify the directories and file names that will be used in the analysis. Edit the following two lines of the runFEL.bf script:

"1": "/path/to/HRH1_aligned_codon.fasta",

                                "2":  "/path/to/RAxML_bestTree.HRH1_tree",
  Here, "1" should specify the full path to the align- ment file HRH1_aligned_codon.fasta, and "2" should specify the full path to the tree file RAxML_bestTree.HRH1_tree.
  Run HyPhy with the following command:

HYPHYMP  runFEL.bf
  An output file HRH1_aligned_codon.fasta.FEL.json is written to the folder that contains the alignment file.
7. Parse HyPhy output ( *see* **Note 3**)
  For further downstream processing, the HyPhy output file in JSON format needs to be converted to CSV format. The custom python script parse_FEL.py will extract the site’s position, *dN* (referred to as ‘beta’ in HyPhy output), *dS* (referred to as ‘alpha’ in HyPhy output), and other site information outputted by HyPhy:

python parse_FEL.py \

                                -j HRH1_aligned_codon.fasta.FEL.json \

                                -r extracted_HRH1_dNdS.csv

  Arguments above correspond to the following:
  • -j, JSON file from the FEL analysis.
  • -r, The output CSV file. If not specified, the output file is site_rates.csv.
8. Calculate site-specific *dN/dS* ( *see* **Notes 3**)
FEL will calculate *dS* and *dN* values for all informative sites. However, the FEL method will assign *dS* = 0 and *dN* = 0 to sites without any synonymous or non-synonymous substitutions, respectively. When calculating *dN/dS* at these entirely conserved sites, we recommend to use the value *dN/dS* = 0. For sites with only one non-gap residue, FEL will similarly assign both *dN* and *dS* a value of 0. For those sites, we also recommend to use the value *dN/dS* = 0.
We provide a custom script that will calculate site-wise *dN/dS* and will assign *dN/dS* = 0 to such sites. For simplicity, this script checks for conserved sites in amino acid alignments. That the script considers amino acid rather than codon conservation does not influence rate assignments, as *dN/dS* = 0 in both cases of fully conserved amino acids and codons. For sites with substitutions, HyPhy’s FEL method, used specifically as recommended here, assigns *dS* = 1. At those sites, our script calculates site-wise *dN/dS* by simply dividing site’s *dN* by site’s *dS*. The original format of extracted_HRH1_dNdS.csv will not be changed.

python calc_dNdS.py \
                  
                                -a HRH1_aligned_aa.fasta \
                  
                                -r extracted_HRH1_dNdS.csv \
                  
                                -o   processed_HRH1_dNdS.csv

Arguments above correspond to the following:
  • -a, The amino acid alignment file.
  • -r, The CSV file with parsed FEL rates.
  • -o, The output CSV file. If not specified, the script outputs processed_dNdS.csv.

### Protocol 3: Measuring structural features

All structural features in this section are calculated from an example PDB file, 3rze.pdb ^30^. This PDB file defines the crystal structure of a human histamine receptor 1 (HRH1), whose rates were computed in Protcols 1 and 2, fused to an unrelated lysozyme protein. The lysozyme is required for crystallization, but is not biologically relevant. We have pre-processed the PDB file to exclude residues from the lysozyme protein (residue numbers 1000 and above). The input and output files used in this section can be found at: https://github.com/clauswilke/proteinER/tree/master/measuring_structural_features.

1. Calculate relative solvent accessibility (RSA) from the PDB file ( *see* **Note 3**)
  We provide a custom script calc_rsa.py that will run the software mkDSSP ^27, 28^, extract absolute solvent accessibilities from the mkDSSP output , and calculate relative solvent accessibilities ^23^. The first argument is the PDB file, and the second op- tional argument ( -o 3rze) is the prefix used for the output files.

python calc_rsa.py 3rze.pdb -o 3rze
  This command will generate two output files: 3rze.asa.txt containing the raw mkDSSP output, and 3rze.rsa.csv containing RSA values and secondary structure classifications.
2. Calculate weighted contact numbers (WCN) from the PDB file ( *see* **Note 3**)
  WCN measures amino acid packing density and may be calculated with respect to either the *α*-carbon or the geometric center of the side-chain ^19, 40^. We provide a custom script that will calculate both types of WCN values, although we strongly recommend using the side-chain WCN values ^19^. The command line arguments follow the same format as the calc_rsa.py script.

python calc_wcn.py 3rze.pdb -o 3rze
  The above command will produce an output file 3rze.wcn.csv that contains both side-chain and *α*-carbon WCN values for each position in the input PDB file.

### Protocol 4: Combining rates with structural features

The input and output files used in this section can be found at: https://github.com/clauswilke/proteinER/ tree/master/map_structural_features.

1. Generate sequence alignment map ( *see* **Note 3**)
  To map site specific evolutionary rates to residues in a PDB structure, we first align the sequence of amino acids extracted from the PDB structure to the multiple sequence alignment used for rate inference. We provide a script that calls MAFFT to align a PDB sequence to a multiple sequence alignment and reformat the output.

python make_map.py \
                   
                                HRH1_aligned.fasta  3rze.pdb
  Running the above command produces a CSV file 3rze.map.csv with four columns. The first and second columns contain the numbered position of a given residue in the *alignment* used for rate inference and the numbered position of a given residue in the *PDB structure*, respectively. The numbered positions in the second column are obtained directly from the PDB input file and may therefore include PDB insertion codes ( *see* **Note 10**). The third and fourth columns contain the single-letter amino acid present in the PDB structure and the PDB chain, respectively. If an amino acid is in the alignment but not in the PDB structure, the PDB position is assigned a value of NA. Likewise, if an amino acid is in the PDB structure but not the alignment, the alignment position is assigned NA.
2. Map rates to structural features ( *see* **Note 3**)
  After mapping the alignment used for rate inference to the sequence of the PDB structure, we merge rates with structural features for each residue. We provide a script that uses the map generated above to combine rates and structural features into a single CSV.

python map_features.py 3rze.map.csv \

                                -r processed_HRH1_dNdS.csv \ extracted_HRH1_rates.csv \

                                -f 3rze.rsa.csv 3rze.wcn.csv

**Figure 1.**
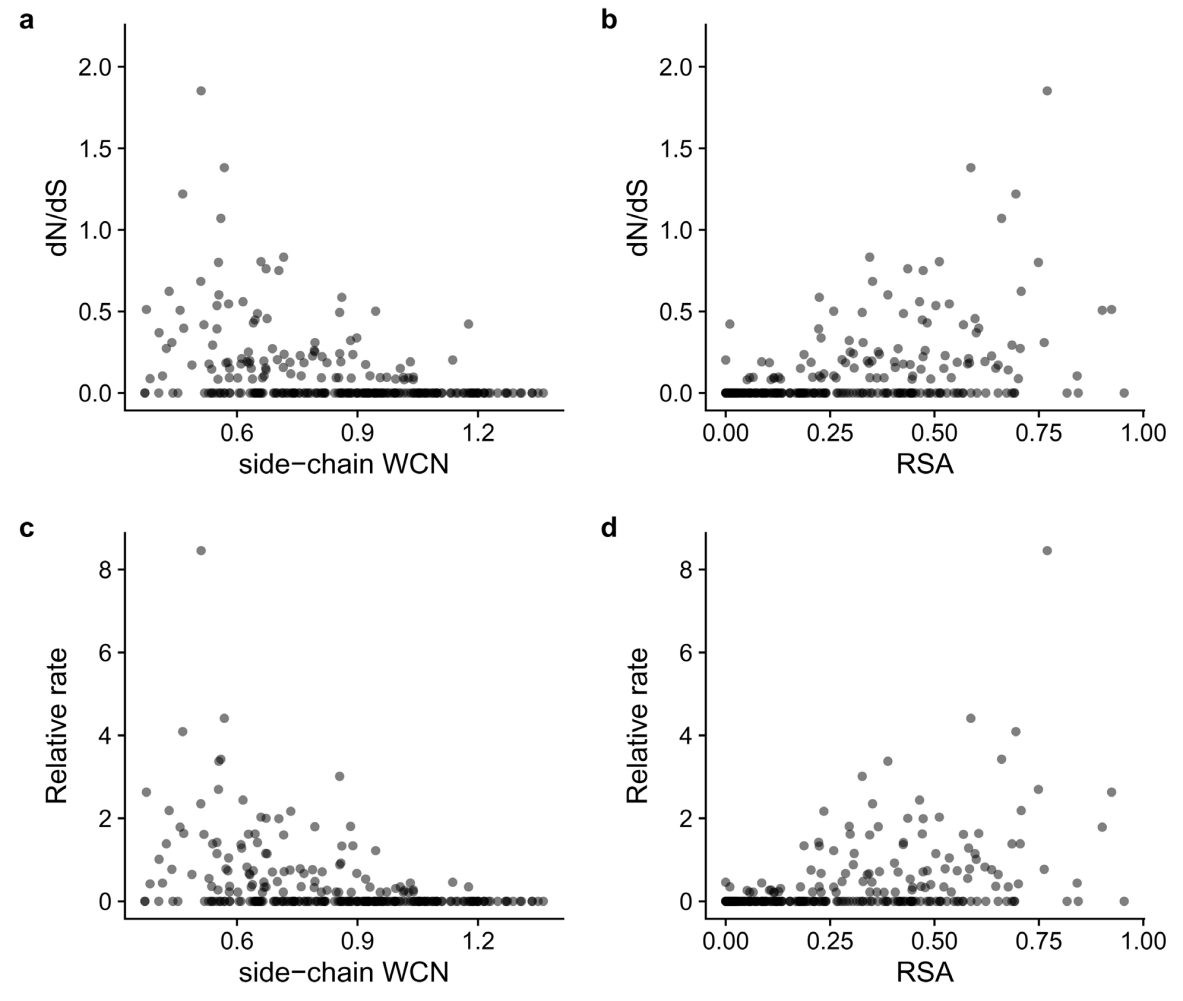
Amino-acid packing density and solvent accessibility correlate with site specific evolutionary rates. ( **a**– **d**) Each point represents a residue in the structure of the HRH1 protein (PDB: 3rze). The Pearson correlation coefficients *r* between structural features (RSA or WCN) and rates ( *dN*/ *dS* or amino acid) are as follows for each panel: ( **a**) –0.39, ( **b**) 0.43, ( **c**) –0.39, ( **d**) 0.42.

  Arguments above correspond to the following:
  • The input file containing a map between the alignment residue positions and the structure residue positions.
  • -r, The rates files.
  • -f, The structural feature files.
  • -o, The CSV output file. If not specified, the script outputs a file <pdb_id>.rates_features.csv. Here, <pdb_id> is the name of the PDB ID used to make the map file.
  The output from this command provides all the data needed to compute correlations between rates and structural features and corresponding visualizations, as, for example, shown in Figure 1.

## Conclusions

We have provided four separate protocols that jointly enable the analysis of protein evolutionary rates in a structural context. The first two protocols measure site-specific evolutionary rates from multiple-sequence alignments, either at the amino-acid or the codon level. In practice, a given study will generally employ only one of these two protocols. The third protocol quantifies local characteristic of a protein structure, such as relative solvent accessibility or weighted contact number, and the fourth protocol maps the structural quantities and evolutionary rates to one another.

In the first two protocols we used two different methods to calculate evolutionary rates, HyPhy’s FEL and LEISR approaches. As an alternative method to measuring amino acid evolutionary rates with LEISR, we presented a brief pipeline in the notes that uses Rate4Site software. Other methods for calculating evolutionary rates have been provided by Rodrigue *et al*. and Tamuri *et al*. ^41– 43^, whose methods infer codon-level site-wise evolutionary rates in a population genetics framework. Other relevant works by Jones at al. and by Halpern and Bruno cover theoretical approaches to infer site-wise rates ^44, 45^.

In sum, we hope that the protocols presented here will be useful for further research into disentangling structural and functional constraints on protein evolution.

## Notes

1. The minimum required HyPhy version for FEL *dN/dS* inference is 2.3.3. The minimum required version for relative amino-acid rate inference with LEISR is 2.3.8. In addition, for users who feel more comfortable working in a python scripting environment than with HyPhy directly from the command line, we note that users can accomplish all HyPhy analyses described here (including parsing HyPhy output) through the package “phyphy” ^46^, available from https://github.com/sjspielman/phyphy.
2. To thread RAxML, compile the raxmlHPC-PTHREADS-SSE3 executable with Makefile.SSE3.PTHREADS.gcc or Makefile.SSE3.PTHREADS.mac. The options to call RAxML stay the same. Add the option -T to thread, and run RAxML with

raxmlHPC-PTHREADS-SSE3 -T 48 \
                  
                            -s HRH1_aligned.fasta \
                  
                            -n HRH1_tree \
                  
                            -m PROTCATLG \
                  
                            -p 12345
3. All of our custom python scripts provide documentation when called with the options -h or --help. For example, to view the documentation for the script calc_rsa.py, run the command
python calc_rsa.py -h
  The script’s use and required input files will be described in the documentation. Additionally, where applicable, the documentation also provides a description of the information stored in the output files.
4. HyPhy will not properly read data if either a pipe character ("|") or a period (".") is present in the input alignment/phylogeny sequence IDs. We recommend to change these characters, consistently in both the alignment and phylogeny, to "_", which HyPhy does accept. We provide a custom python script to execute this step in the alignment, which will in turn propagate to a tree reconstructed from this alignment:

python format_aln_id.py \
         
                            -a HRH1_aligned.fasta\
         
                            -o HRH1_aligned_reformatted.fasta

  Arguments above correspond to the following:
  • -a, The input file containing sequences in the FASTA format. Both aligned and aligned sequences are accepted.
  • -o, The output file with reformatted sequence IDs. If not specified, the output file is reformatted_aln.fasta.
5. RAxML can also infer trees from nucleotide sequence data in addition to amino-acid data. Importantly, if the analyzed sequences don’t show much divergence at the amino-acid level, then trees inferred from nucleotide sequences may yield better rate predictions. To infer a tree from nucleotide data with RAxML, issue the following command (specifically, -m PROTCATLG has been changed to a GTR nucleotide model with CAT heterogeneity, -m GTRCAT):

raxmlHPC-SSE3 -s HRH1_aligned.fasta \
                
                            -n HRH1_tree -m GTRCAT \
                
                            -p 12345

  Furthermore, if the dataset of interest contains fewer than 50 taxa, a discrete Gamma distribution should be used rather than the CAT model for modeling rate heterogeneity ^38^. To specify this model, simply replace the phrase CAT with GAMMA: For amino-acids, use the model specification -m PROTGAMMALG, and for nucleotides use the model specification -m GTRGAMMA.
6. As an alternative method to infer site-wise amino acid rates one can use Rate4Site (or its accompanying webserver, ConSurf ^47^). Rate4Site is a tool for inferring site-wise evolutionary rates in amino acid sequences ^20^. Download Rate4Site from https://www.tau.ac.il/~itaymay/cp/rate4site.html. Analyses presented here use Rate4Site downloaded as rate4site.3.2.source.zip and compiled with the Makefile_slow file.
  LEISR, in fact, is based on the Rate4Site algorithm, and these approaches therefore produce virtually identical rates ^39^. However, LEISR provides increased functionality relative to Rate4Site, namely by assigning rates to all alignment positions and by allowing for datasets of arbitrary size. In addition, Rate4Site normalizes rates to standard z-scores, whereas LEISR performs no such normalization. Finally, Rate4Site is available both as a random-effects and fixed-effects implementation, and LEISR adopts the fixed-effects approach. The fixed-effects implementation may be preferable, because random-effects models shrink and/or smooth rate estimates, which can produce undesirable artifacts in the inferred rates.
  The options to run Rate4Site may be different for different Rate4Site installation files. We recommend using the rate4site -h command to find the proper options for your version, as opposed to using the software’s website.
  Run the following command to infer site-wise rates:

rate4site -Mw -s HRH1_aligned.fasta \
          
                            -t RAxML_bestTree.HRH1_tree \
          
                            -o HRH1_norm_rates.txt \
          
                            -y HRH1_orig_rates.txt

  Arguments above correspond to the following:
  • -Mw, Specify the WAG model of amino-acid evolution ( *see* **Note 9**).
  • -s, The multiple sequence alignment file.
  • -t, The input phylogeny.
  • -o, The output file of *normalized* amino-acid rates.
  • -y, The output file of *raw* amino-acid rates.
  Rate4Site normalizes rates by converting them into standard z-scores. The z-scores are written to HRH1_norm_rates.txt. Rate4site also outputs the raw (unnormalized) scores in HRH1_orig_rates.txt. We advise you to use raw scores and to normalize them by the average score in the sequence, as discussed in protocol 1 step 6. Note that Rate4Site also outputs a new tree file TheTree.txt and an empty rates file r4s.res. These files are not needed for further analysis.
  For further downstream processing, the Rate4Site output file needs to be converted to a CSV file. The following command will extract the site’s position, amino acid, and Rate4Site score ( *see* **Note 3**).

python parse_r4s.py \
        
                            HRH1_orig_rates.txt \
        
                            -o extracted_HRH1_orig_rates.csv
  Arguments above correspond to the following:
  • The Rate4Site output file.
  • -o, The output CSV file name.
  By default, the Rate4Site software will compute rates only for the sites in the first sequence of the alignment file. In other words, Rate4Site will ignore any alignment columns where the site in the first sequence is a gap. To circumvent losing information outputted from Rate4Site, we suggest finding the sequence in the alignment with the fewest gaps and using it as the reference sequence for the output. The reference sequence for Rate4Site can be specified with the option -a sequence_ID, where sequence_ID is the name of the sequence in a FASTA file provided for rate inference.
7. If you are interested in calculating relative rates in R, our script make_plots.R contains code to normalize the rates to the mean of 1. This script reads in the last output file from protocol 4 to plot Figure 1. Prior to plotting, the script will normalize rates relative to the gene-wide average.
8. The file runFEL.bf implements fixed-effect likelihood (FEL) inference without synonymous rate variation, which is sometimes referred to as a one-rate FEL model. This parameterization infers one *dN* value per site and one *dS* value for the entire sequence ^21^. The one-rate FEL model has been found to infer more accurate *dN/dS* values than models which infer a separate *dS* at each site ^22^.
9. For reasons that are beyond the scope of this paper, the specific matrix choice has little effect on the final rates, as long as rates are normalized relative to their means as we do here. The underlying reason for this insensitivity to matrix choice is that the available matrices were all derived by pooling data from many sites in many proteins (see e.g. 37) , and this pooling yields matrices that are close to uninformative ^48, 49^.
10. The residue numbers in PDB files are not strictly sequential or numeric. If multiple residues share the same numeric value, they will be distinguished by a single letter insertion code (e.g. 53A or 53B) ^50^. These insertion codes appear when there are several homologous proteins with crystal structures. Generally, each new structure retains the numbering of the earliest crystallized structure to preserve the alignment among structures of homologous proteins. If the new structure contains deletions relative to the original structure, the PDB file will skip residue numbers. If the new structure contains insertions, the PDB file will have residue numbers with insertion codes.

## Data and software availability

All information required to reproduce the analysis is provided at https://github.com/clauswilke/proteinER. Version 2.0 of this code is archived at https://doi.org/10.5281/zenodo.1160661.
