# Supplementary material for: Measuring evolutionary rates of proteins in a structural context
Source: F1000Res. 2018 Feb 9;6:1845. Originally published 2017 Oct 16. [Version 2] doi: 10.12688/f1000research.12874.2 (PMC5676193; doi:10.12688/f1000research.12874.2)
Supplement: Peer review history

# Peer review: Referee response for version 2

**DOI:** 10.5256/f1000research.15080.r30718

**Published:** 2018-02-12

**Authors:** Yu Xia, Avital Sharir-Ivry

**Affiliations:** 1 Department of Bioengineering, Faculty of Engineering, McGill University, Montreal, QC, Canada

All comments have been adequately addressed.

We have read this submission. We believe that we have an appropriate level of expertise to confirm that it is of an acceptable scientific standard.

# Peer review: Referee response for version 1

**DOI:** 10.5256/f1000research.13954.r27040

**Published:** 2017-11-17

**Authors:** Mario dos Reis

**Affiliations:** 1 School of Biological and Chemical Sciences, Queen Mary University of London, London, UK

This paper by Sydykova *et al.* is written in tutorial style, with the aim of demonstrating how to use a series of software tools to calculate site-specific rates of evolution in proteins and protein-coding sequences. In brief, the user starts with a molecular sequence alignment as input data, and then proceeds to estimate a phylogenetic tree, site-specific evolutionary rates, and other statistics related to protein structure and evolution.

 The things I look for in a good tutorial are clarity and brevity. This manuscript achieves both. Brevity is important as, in my opinion, a tutorial user should not be bogged down with lengthy discussions of theory, caveats, comparisons to other methods, etc. However, what I do expect from a good tutorial is a set of key sentences pointing out to the user where to find the appropriate theory papers, simulation studies, and work by others that may have developed similar methods.

 I have the following recommendations:

- Perhaps at the beginning of the protocol section, the authors could clearly state to the user what level of knowledge is expected from them. Does the user need to know how to use the command line? R? Must they have some basic experience of using phylogenetic software? This is important, specially in this case, where the user base may come from a very mixed background (physical sciences, computer science, chemistry or biology).
- The authors may consider adding a section at the end of the paper with general recommendation for this type of analysis, and where they point the user towards additional literature methods. By putting this section at the end, the user is not bogged down with this information in the middle of the tutorial. For example, Yu Xia recommends mentioning the ConSurf server. This appears a reasonable request. I think the authors should also consider mentioning here other related methods, as this will be useful information to the user. For example, the works by Rodrigue *et al*. ^1^^-^^2^ and Tamuri *et al*. ^3^ could be mentioned in reference to population genetic ways to measure site-specific rates and dN/dS. It may also be desirable to cite some of the more modern theoretical works on this, for example Halpern and Bruno ^4^, Jones *et al*. ^5^, etc. These are just suggestions. Similarly, Ugo Bastolla suggests adding some mention of what the results would like under other software, for example, in comparison with Rate4site. Although an extensive analysis of this topic may seen out of the scope of the tutorial, I think a set of references from the literature would be in order.
- Finally, I think the tutorial would benefit with the inclusion of a few additional figures. For example, the authors may want to consider adding a histogram of dN/dS vs. protein site, and perhaps also add screenshots of key program outputs. A well-illustrated tutorial is more appealing to students. Screenshots of program output are essential as it allows the user to check whether they are indeed obtaining the correct results. Perhaps also include tables summarising result values for key parameters.

I have read this submission. I believe that I have an appropriate level of expertise to confirm that it is of an acceptable scientific standard.

## References

[1] RodrigueNPhilippeHLartillotN: Mutation-selection models of coding sequence evolution with site-heterogeneous amino acid fitness profiles. *Proc Natl Acad Sci U S A*.2010;107(10) : 10.1073/pnas.0910915107 4629-34 10.1073/pnas.0910915107 20176949PMC2842053

[2] RodrigueNLartillotN: Site-heterogeneous mutation-selection models within the PhyloBayes-MPI package. *Bioinformatics*.2014;30(7) : 10.1093/bioinformatics/btt729 1020-1 10.1093/bioinformatics/btt729 24351710PMC3967107

[3] TamuriAUdos ReisMGoldsteinRA: Estimating the distribution of selection coefficients from phylogenetic data using sitewise mutation-selection models. *Genetics*.2012;190(3) : 10.1534/genetics.111.136432 1101-15 10.1534/genetics.111.136432 22209901PMC3296245

[4] HalpernABrunoW: Evolutionary distances for protein-coding sequences: modeling site- specific residue frequencies. *Molecular Biology and Evolution*.1998;15(7) : 10.1093/oxfordjournals.molbev.a025995 910-917 10.1093/oxfordjournals.molbev.a025995 9656490

[5] JonesCTYoussefNSuskoEBielawskiJP: Shifting Balance on a Static Mutation-Selection Landscape: A Novel Scenario of Positive Selection. *Mol Biol Evol*.2017;34(2) : 10.1093/molbev/msw237 391-407 10.1093/molbev/msw237 28110273

# Response

**Published:** 2018-01-26

**Authors:** Claus Wilke

**Affiliations:** The University of Texas at Austin, USA

Dear Dr. Mario dos Reis,

We thank you for your helpful comments and suggestions. We have added additional notes, where appropriate, to indicate other theory papers, methods, and simulations a user might find useful. In the note about Rate4Site, we have added a brief paragraph that discusses the differences between Rate4Site and HyPhy’s LEISR method. We have also added a paragraph in the Conclusions describing other relevant works. In that paragraph, we have cited the papers you suggested.

In the protocols section, we have added some information about the level of skill needed to run our analysis. We have replaced “Throughout, we assume that we are working on a UNIX-like command line interface” with the following: “Throughout, we assume that we are working on UNIX-like command line interface. We recommend that a user is comfortable with command execution and syntax, which includes flags, arguments, and directories. No prior knowledge of any of the listed software is essential. For python and R scripts, we provide detailed description for each script’s function. As such, it is not strictly necessary that a user knows python or R to execute our pipeline. However, if more detailed understanding of the custom scripts is desired, a user should be familiar with python and R.”

We also wanted to address your comment to include screenshots with program outputs. We have provided the git repository for this purpose. The git repository contains all input and output files the pipeline has generated. We feel that this is sufficient for the user to check their execution of our pipeline. A user can compare the output files we have provided to the output files he or she generated. We also have provided detailed descriptions of the output files for each step. We believe those should be enough to assess if the software has run and whether it has run correctly.

# Peer review: Referee response for version 1

**DOI:** 10.5256/f1000research.13954.r27041

**Published:** 2017-11-07

**Authors:** David D Pollock

**Affiliations:** 1 Department of Biochemistry & Molecular Genetics, University of Colorado School of Medicine, Aurora, Colorado, 80045, USA

The other two reviewers did a good job, such that I am going to be more philosophical about my review by focusing on two questions: Is this the best format for this kind of information, and am I (or the other two reviewers) the right person to review it? Other than a brief introduction and conclusion, the manuscript is mostly a detailed recipe of a specific pathway to assess correlations between evolutionary rates and some crystal structural features (when such information is available). This might be useful to a range of users such as undergraduates and beginning graduate students for whom it would serve as a gateway to thinking about these kinds of questions. The described pipeline is not a substantive modification to existing methods or an innovative application to to new scientific questions, but rather is a tool designed to facilitate performance of experiments in a particular way. I would hope that more advanced students would quickly want to modify the experiments and tools that make up this pipeline, asking questions about the assumptions that the included tools make, and asking questions about alternative methods of analysis. Should one use only a maximum likelihood “best tree”, or should one consider a bootstrap or Bayesian posterior distribution? How does model choice interact with inference of site-specific rates? What assumptions were made in the alignment, and do those assumptions and errors in the alignment interact with the phylogenetic and rates inferences? In many ways, rather than a fixed and published document, the author’s pipeline might be better implemented in the context of an interactive and perhaps continuously modified lesson plan to teach students about the underlying issues, with more direction and encouragement to substitute in different methods and models. This leads to the question of whether I, or other professors, are the right people to review this. I trust Wilke and his laboratory members to have tested and made this pipeline function, and I don’t have time in the context of reviewing a paper to install and implement and test their scripts. But what they and I and other professors or advanced students are going to be bad at anyway is figuring out how hard it is and how it can go wrong in the hands of beginning students. So my suggestion is that it is beginning students who should be reviewing this pipeline, trying to implement it and trying to break it (or breaking it without trying). That would seem to me to be a more meaningful review.

 As an aside, publishing answers to simplistic bubble questions seems inane to me, especially without the choice to opt out, and that is the context in which they were filled.

I have read this submission. I believe that I have an appropriate level of expertise to confirm that it is of an acceptable scientific standard.

# Response

**Published:** 2018-01-26

**Authors:** Claus Wilke

**Affiliations:** The University of Texas at Austin, USA

Dear Dr. David Pollock,

We thank you for your comments and ideas. For specific feedback, you referred us to the other reviewers, and we have addressed their comments and modified our paper where appropriate.

# Peer review: Referee response for version 1

**DOI:** 10.5256/f1000research.13954.r27042

**Published:** 2017-11-06

**Authors:** Yu Xia, Avital Sharir-Ivry

**Affiliations:** 1 Department of Bioengineering, Faculty of Engineering, McGill University, Montreal, QC, Canada

This paper provides a complete and detailed recipe for calculating evolutionary rates and structural features of residues in proteins, and for correlating site-specific evolutionary rates with structural features.  Such analysis is important for better understanding of protein structure-evolution relationships at the residue level.  This paper describes all the computational steps in detail including the required computer programs and their installation, provides scripts to run the different programs and to process the results, and introduces a repository with all the scripts.  This paper very clearly explains the steps needed to fully perform such analyses.  The computational pipeline provided is expected to become a useful resource to the scientific community.  This paper could potentially benefit from addressing a few minor issues described below.

 1. While the paper aims to describe a complete analysis, the initial step of how to properly collect protein sequences for subsequent evolutionary analyses is not described.  For the sake of completeness and to help interested readers to perform such analyses from scratch, it will be beneficial to at least mention this step (and preferably provide a more detailed description).

 2. A full and automated procedure for analyzing relative amino acid rates already exists with the ConSurf server.  At the same time, the second protocol described in this paper can be especially useful for site-specific codon rate calculations, which is not included in ConSurf.  It will be beneficial to provide more context regarding other available procedures and how the procedure described in this paper complements these other available procedures.

 3. It will be beneficial to include the scripts for normalizing the inferred relative rates as well as for finding the sequence in the MSA with smallest number of gaps.  These steps are described in the paper but it appears that the corresponding scripts are not included.

 4. The file name of the output for Step 1 of Protocol 4 (pdb_id.map.csv) is not described in the text.

 5. It is unclear how the order of the lines in the output pdb_id.rates_features.csv is decided.  Because this is the final output of the entire analysis, it will be useful to organize this output as clearly as possible.

We have read this submission. We believe that we have an appropriate level of expertise to confirm that it is of an acceptable scientific standard.

# Response

**Published:** 2018-01-26

**Authors:** Claus Wilke

**Affiliations:** The University of Texas at Austin, USA

Dear Drs. Yu Xia and Avital Sharir-Ivry,

We thank you for your helpful comments and suggestions. We have added an additional step about collecting homologous sequences to both protocols 1 and 2. We also added a paragraph in the Conclusions about other methods to infer site-wise evolutionary rates. We also now discuss the differences between the different methods. See also our response to Dr. Mario dos Reis’s comments. 

Further, we now provide the name of the output file for step 1 of protocol 4, and we have added a note to step 6 of protocol 1 to state that our script to plot the figure contains code to normalize inferred rates. When we worked on version 1 of our publication, HyPhy’s LEISR method was not yet published. Both Rate4Site and LEISR infer similar rates. We encourage users to use LEISR over Rate4Site to circumvent little issues Rate4Site has, such as not outputting site-wise rates for all site in the alignment.

We have refrained from organizing the order of rows in the output file pdb_id.rates_features.csv. There are two columns by which we could order: the site position of the protein structure or the site position of the alignment. Because the sequence of the PDB structure is typically different from the ones in the alignment, the two will not align perfectly to each other. This introduces gaps in either of the two or both sequences. It is, therefore, unclear what the order of lines with gaps should be.

# Peer review: Referee response for version 1

**DOI:** 10.5256/f1000research.13954.r27048

**Published:** 2017-11-02

**Authors:** Ugo Bastolla

**Affiliations:** 1 Severo Ochoa Molecular Biology Centre (CSIC-UAM), Autonomous University of Madrid, Madrid, Spain

The structural properties of a protein site in the native state of the protein are known to strongly influence its evolutionary rate, according to research of which the corresponding author has been a prominent exponent. This finding gives useful insights on how natural selection targets the structural properties of proteins.

 This paper presents computational protocols for measuring site-specific substitution rates in protein families, either at the amino-acid or at the codon level, and mapping them to the site-specific structural descriptors that have been found to influence the rates most strongly, such as relative solvent accessibility and weighted contact number. All the necessary software is clearly illustrated and all the relevant input and output files are made available to researchers willing to repeat the exercise, to whom this report will be for sure useful.

 However, I think that the paper falls a bit short in motivating why it is interesting to perform this computation for a protein family of interest, besides recovering the known correlations between evolutionary rates and structural descriptors. Secondly, different methods and software exist to compute substitution rates. The authors show that, for the protein family that they choose, rates at the amino-acid and at the nucleotide level obtained with the same software yield almost identical results. How do results compare using different software (Rate4site versus HyPhy)? How do the authors interpret the sites with estimated dN/dS>1, which is considered an indication of positive selection, in the context of the protein structure?

I have read this submission. I believe that I have an appropriate level of expertise to confirm that it is of an acceptable scientific standard.

# Response

**Published:** 2018-01-26

**Authors:** Claus Wilke

**Affiliations:** The University of Texas at Austin, USA

Dear Dr. Ugo Bastolla,

We thank you for your helpful comments and suggestions. We now cite the new LEISR paper that demonstrates that Rate4Site and HyPhy’s method LEISR produce similar rates. We reference this paper in the note about Rate4Site.
